# Stressful life events, psychosocial health and general health in preschool children before age 4

**DOI:** 10.1007/s12519-022-00639-w

**Published:** 2022-11-17

**Authors:** Jie Luo, Amy van Grieken, Shuang Zhou, Yuan Fang, Hein Raat

**Affiliations:** 1 Department of Public Health, Erasmus MC, Dr. Molewaterplein 40, 3015 GD Rotterdam, The Netherlands; 2 Department of Maternal and Child Health, School of Public Health, Peking University, Beijing, China

**Keywords:** Child health, Preschool children, Stressful life event

## Abstract

**Background:**

The impact of stressful life events (SLEs) in early childhood is often ignored. We aimed to examine longitudinal associations between SLEs and psychosocial and general health in preschool children.

**Methods:**

Twelve SLEs occurring before the age of 24 months were assessed and categorized by frequency (no events, 1–2 SLEs, and > 2 SLEs) and overall tension (no events, low, and high) (*n* = 1431). Psychosocial and general health were measured three times at the age of 24, 36 and 45 months. The associations were examined by logistic regression models using generalized estimating equations to handle repeated measurements.

**Results:**

Half (48.4%) of the families experienced SLEs, and 23.8% perceived high-tension SLEs before the children were aged 24 months. Gender differences were observed in the association between SLEs and psychosocial health. Compared to girls without SLEs, girls who experienced > 2 SLEs [OR = 3.31, 95% confidence interval (CI) 2.05–5.35] or high-tension SLEs (OR = 3.01, 95% CI 2.07–4.39) had higher odds of psychosocial problems from 24 to 45 months. The odds ratios in boys were 2.10 (95% CI 1.36–3.24) and 1.47 (95% CI 1.06–2.03), respectively. Moreover, only girls’ risk of psychosocial problems increased after experiencing 1–2 SLEs (OR = 2.15, 95% CI 1.54–3.00) or low-tension SLEs (OR = 1.90, 95% CI 1.31–2.74). Regarding general health, children who experienced > 2 SLEs (OR = 1.96, 95% CI 1.21–3.18) and high-tension SLEs (OR = 1.60, 95% CI 1.12–2.28) had higher odds of poor general health from 24 to 45 months.

**Conclusions:**

The findings emphasized that young children’s psychosocial and general health can be impacted by experiencing SLEs in early childhood. Attention and adequate support for families experiencing SLEs are needed to minimize the potential negative effect of SLEs on child health, particularly in girls.

**Supplementary Information:**

The online version contains supplementary material available at 10.1007/s12519-022-00639-w.

## Introduction

Stressful life events (SLEs) refer to any threat that might cause behavioral/physiological responses and subsequent development of diseases [1]. SLEs encompass various experiences and exposures, e.g., divorce, neglect, financial difficulty, and death of family member(s)/close friend(s) [2]. Previous work highlights that childhood SLEs are associated with poor cognitive function and school achievement in adolescence [3, 4]. From a life-course perspective, SLEs are strongly associated with various health outcomes during the lifetime, including substance abuse, chronic physical conditions (e.g., respiratory disease and metabolic disease), and psychosocial conditions (e.g., depression and anxiety) [5–7].

Worldwide interest is growing in health development in early childhood, both the first 1000 days of life (conception to age two) and the next 1000 days (from age 2 to school entry) following a life-course approach [8]. Psychosocial health consists of emotional, behavioral and social well-being. Children with psychosocial problems in early childhood are more likely to have learning difficulties, substance use and mental health diseases in adulthood [9].

Although there are extensive studies on SLEs and psychosocial health, most have been conducted in adolescence and adulthood [4–7, 10]. In a systematic review on early-life stress and depression in children (0–18 years), among 62 studies, the study with the youngest sample reported upon children aged 7.75 years [7]. Adverse exposures in early childhood, such as SLEs, may harm individuals up to adolescence [9, 11]. For example, Nishikawa et al. observed that high school students exposed to SLEs before primary school had higher levels of stress intensity and posttraumatic stress symptoms [12]. On the positive side, timely evaluation of the impact and effective interventions in early childhood also showed greater benefit to individual and societal outcomes [5, 11].

Previous studies noticed an accumulative effect of experiencing multiple SLEs on eating problems and risk behavior [4, 13]. The latest longitudinal study by Martin-Herz reported that the cooccurrence of multiple SLEs could predict behavioral problems in preschool children [14]. This accumulative effect might also be present in other health conditions leading to poor health outcomes in preschool children who experienced multiple SLEs [2, 6]. In addition to the frequency of SLEs, the perceived tension caused by SLEs might differ between individuals [15]. In addition, gender has been reported to moderate the association between stress and child health, especially psychosocial health [2, 16]. Specifically, the types and severity of health outcomes could differ between boys and girls when responding to SLEs [16].

Therefore, this paper aimed to evaluate the longitudinal associations between SLEs occurring before the child’s age of 24 months and psychosocial health and general health at the ages of 24, 36 and 45 months. Specifically, we aimed to (1) examine the association between SLEs and psychosocial health; (2) examine the association between SLEs and general health, and (3) explore whether child gender moderates the associations. We hypothesized that children who experienced more SLEs and higher tension of SLEs would have higher risks of lower health.

## Methods

### Study design and population

A longitudinal design was applied using data from a cohort study. In 2014 and 2015, 2305 parents in the Rotterdam-Rijnmond visited Dutch Preventive Youth Health Care (YHC) and submitted the completed questionnaires at the child’s age of 24 months. At two follow-ups, data were available for 1540 and 1257 children at the ages of 36 months and 45 months, respectively. More details can be found elsewhere [17]. Regarding the information on children, we excluded cases with missing data on stressful life events (*n* = 86), with only one measurement of psychosocial problems, general health, or both (*n* = 639), and with missing data on potential confounders (*n* = 149). In addition, only one child of a twin was included due to the shared family SLEs (*n* = 18). Finally, 1431 children were included in the population for analyses in this study (Supplementary Fig. 1). The Medical Ethical Committee of the Erasmus Medical Center Rotterdam has reviewed the research proposal and declared that the rules laid down in the Medical Research Involving Human Subjects Act (also known by its Dutch abbreviation WMO) did not apply to this research proposal (number MEC-2014–152). This study was conducted by following the guidelines proposed in the World Medical Association Declaration of Helsinki. All participants provided written informed consent to participate in the study.

### Measurements

#### Stressful life events

When children were 24 months old, parents were asked if SLEs had occurred in the past 24 months through parent-reported questionnaires. A list of 12 SLEs was provided, adapted from the Adverse Life Events Scale [18]: relocation of the family; relocation of someone close to the child; tensions felt at home from work; unemployment of one of the parents; financial problems; quarrels with neighbors/friends/acquaintances/family; problems within relationship of parents; divorce; victim of burglary or fire; physical health problems of someone close to the family; mental health problems of someone close to the family; death of someone close to the family. The correlation between the SLEs was low (Spearman correlation coefficient = 0.39). If parents confirmed that a SLE had occurred in the past 24 months, they were asked to specify to what extent this caused stress or tension using a 3-point scale (1 = a little, 2 = somewhat, 3 = a lot).

For the current analyses, two variables regarding the frequency and tension of SLEs were generated. The “frequency of SLEs” was computed by summing up the frequency of confirmed SLEs (ranging from 0 to 12) and classified into three groups: no SLEs, 1–2 SLEs or > 2 SLEs according to the frequency distribution. Based on the tension experienced from SLEs, a variable “overall tension” from SLEs was created and classified into three groups: (a) “no event” (no experience of SLEs, considered the reference group); (b) “low” tension (participants of whom all experienced SLEs had a tension score of 1), and (c) “high” tension (participants who reported at least one experienced life event with a tension score of 2 or 3). Supplementary Table 1 presents the frequency and tension scores per individual life event. Supplementary Table 2 presents a cross-tabulation between frequency and tension scores.

#### Psychosocial health

Psychosocial health was measured by the Brief Infant-Toddler Social–Emotional Assessment (BITSEA) in children aged 24 months and by the Strengths and Difficulties Questionnaire (SDQ) in children aged 36 and 45 months. The reliability and validity of both tools is considered good, also in the Dutch population [19, 20].

The BITSEA is specifically designed to measure psychosocial problems in children aged 12 to 36 months and was used to assess psychosocial health at 24 months [21]. There are 31 items in Problem scale and 11 items in the Competence scale. Each item is scored 0 for “not true”, 1 for “somewhat true”, and 2 for “certainly true”. Scores within each scale were summed, and two scores were generated: “score in Problem scale” and “score in the Competence scale”. Children having at-risk scores on the Problem scale (a 14), Competence scale (s 15), or both were categorized as “yes” at risk of psychosocial problems. The remaining children were categorized as having “no” risk of psychosocial problems.

The SDQ was used for children aged 36 and 45 months. It is a brief questionnaire measuring psychosocial problems for children aged 3–16 years [22]. It consists of five five-item scales: emotional symptoms, conduct problems, hyperactivity-inattention, peer problems, and prosocial behavior [22]. A total difficulty score is calculated by summing the scores of the first four scales (range 0–40). Children with subclinical (9–11) or clinical scores (12–40) in the total difficulty score were categorized as “yes” at risk of psychosocial problems. Children with total difficulty scores under the (sub)clinical cutoffs were categorized as having “no” risk of psychosocial problems. Cutoff scores for both BITSEA and SDQ were based on the reference value in the Netherlands [19, 20]. Participants who had at least two out of three measurements of psychosocial health were included in the analyses (*n* = 1428 at 24 months, *n* = 1331 at 36 months, and *n* = 1085 at 45 months).

#### General health

Children’s general health (good vs. poor) was measured by the Infant Toddler Quality of Life Questionnaire (ITQOL) at 24, 36, and 45 months. The ITQOL is a 47-item tool for infants and toddlers from two months to five years of age [23]. The Global Health scale and General Health scale were used to calculate a general health score. The raw score of each item in the two scales was transferred into 0 (lowest) to 100 (highest level of health); then, the mean score of all items was calculated [24]. Children with a mean score lower than 61.7 were categorized as having “poor” general health. The remainder with a score above 61.7 was categorized as having “good” general health. Participants who had at least two out of three measurements of general health could be included in the analysis population. The available participants for general health were 1423 aged 24 months, 1340 aged 36 months, and 1101 aged 45 months.

#### Other measurements

Several child and parental characteristics were measured: child’s age, gender, ethnic background, respondent of baseline questionnaire (mother/father), maternal age, maternal ethnic background, maternal education level, paternal age, paternal ethnic background, paternal education level, and single-parent family (yes/no). Child age (in months) was measured three times, and the other characteristics were obtained by the baseline questionnaire. The ethnic background of the parents and the child (Dutch/non-Dutch) was defined by the country of birth of their parents according to the Classification of Statistics Netherlands [25]. When both parents were born in the Netherlands, the person was considered to have a Dutch background. When either one or both parents were born outside the Netherlands, the person was considered to have a non-Dutch background. Parental education level was categorized as high, middle, or low following the Classification of Statistics Netherlands [26]. A single-parent family (yes/no) was considered to be “yes” when the child lived with either the father or the mother.

### Statistical analysis

Descriptive statistics were used to describe the sociodemographic characteristics and child health characteristics. One-way analysis of variance (ANOVA) for continuous variables and Chi-square tests for categorical variables were adopted to examine the statistically significant differences between subgroups by frequency of SLEs in Table 1. A *P* value of < 0.05 was considered to indicate statistical significance.

**Table 1:** Sociodemographic characteristics of the study population

Variables	Total sample (*n* = 1431)	Frequency of SLEs			*P* value
		No life events (*n* = 739)	1–2 SLEs (*n* = 546)	> 2 SLEs (*n* = 146)	
Child age at baseline (mon)	24.5 ± 1.8	24.4 ± 1.7	24.5 ± 2.1	24.5 ± 1.7	0.676
Child age at the 1st follow-up (mon)	35.1 ± 2.6	35.0 ± 2.4	35.1 ± 2.8	35.0 ± 2.6	0.760
Child age at the 2nd follow-up (mon)	44.2 ± 3.1	44.1 ± 2.9	44.3 ± 3.2	44.1 ± 3.2	0.806
Child gender, boy	712 (49.8)	376 (50.9)	266 (48.7)	70 (47.9)	0.670
Child ethnic background, Dutch	1160 (81.1)	601 (81.3)	439 (80.4)	120(82.2)	0.857
Mother age (y)	33.1 ± 4.6	33.6 ± 4.7	32.9 ± 4.3	31.9 ± 4.8	< 0.001
Maternal ethnic background, Dutch	1059 (75.9)	552 (76.8)	396 (74.4)	111(76.6)	0.621
Maternal education level					
High	853 (59.6)	439 (59.4)	335 (61.4)	79 (54.1)	0.488
Middle	498 (34.8)	262 (35.5)	180 (33.0)	56 (38.4)	
Low	80 (5.6)	38 (5.1)	31 (5.7)	11 (7.5)	
Father age (y)	37.4 ± 6.3	36.9 ± 5.9	38.5 ± 7.0	35.4 ± 4.7	0.194
Paternal ethnic background, Dutch	1096 (78.1)	572 (78.9)	418 (78.1)	106 (74.1)	0.451
Paternal education level					
High	730 (52.4)	384 (53.5)	293 (54.9)	53 (37.9)	0.003
Middle	511 (36.7)	256 (35.7)	192 (36.0)	63 (45.0)	
Low	151 (10.8)	78 (10.9)	49 (9.2)	24 (17.1)	
Single-parent family, yes	71 (5.0)	28 (3.8)	22 (4.0)	21 (14.4)	< 0.001

Data presented as Mean ± SD or *n* (%)

Number of missing: maternal ethnic background = 35, paternal ethnic background = 28, paternal education level = 39

*SD* standard deviation, *SLE* stressful life event

*P* values are based on Chi-square test for categorized data or independent *t* test for continuous data

All dependent variables (i.e., psychosocial and general health variables) were measured three times when children were aged 24, 36, and 45 months. Generalized estimating equations (GEEs) to handle the repeated measurements were applied in logistic regression models. First, the independent correlation structure was chosen after comparing which has the highest score of quasi-likelihood under the independence model criterion (QIC), indicating the best model fit for GEE. Second, four crude models were built to examine the association between SLEs and children’s health: (1) frequency of SLEs and psychosocial health; (2) overall tension experienced and psychosocial health; (3) frequency of SLEs and general health; and (4) overall tension experienced and general health. Third, we tested the potential covariates, including child age, child gender, child ethnic background, socioeconomic status (i.e., maternal education level, single-parent family, and neighborhood socioeconomic status), and respondent of the baseline questionnaire by univariate regression. Finally, four models were adjusted for significant covariates, i.e., child age, child gender, child ethnic background, maternal education level, and single-parent family, based on the significant association with two health outcomes (*P* < 0.05). Whether the mother or the father completed the SLEs list and other baseline information did not show a significant difference in child health (*P* > 0.05).

Child gender, age, and ethnic background are the most highlighted moderators in similar association studies [16]. Interaction items of child gender, age, and ethnic background were individually added into four models. After applying Bonferroni correction (*P* = 0.05/3 = 0.017), gender significantly modified the association of both the frequency and overall tension of SLEs with psychosocial problems (both *P* < 0.017). Therefore, stratified results of the frequency and overall tension of SLEs and psychosocial problems are presented. Child age only significantly modified the association between overall tension and psychosocial health (*P* < 0.017). Stratified results by child age are presented in Supplementary Table 3. No moderation was observed for ethnic background.

Excluded children (*n* = 874) were compared to included children (*n* = 1431) with regard to sociodemographic characteristics (Supplementary Table 4). All analyses were completed by IBM SPSS version 28 for Windows (IBM Corp., Armonk, NY, USA).

## Results

### Characteristics of the study population

Table 1 shows the characteristics of 1431 children aged 24.5 [standard deviation (SD) = 1.8] months at baseline. Half were boys (49.8%), most were Dutch (81.1%), and only a few (5.0%) lived in a single-parent family. For parents, more than half had a high education level (mother 59.6%; father 52.4%), and most had a Dutch background (mother 75.9%; father 78.1%). Maternal age, paternal education level and single-parent family showed differences when comparing the three subgroups by the frequency of SLEs (all *P* < 0.05); the remaining demographic variables were independent of the frequency of SLEs (all *P* > 0.05).

Compared to participants excluded in the analyses (*n* = 874), participants included (*n* = 1431) were, as a child, more likely to have a non-Dutch background and live in a single-parent family; as a parent, they were more likely to have a non-Dutch background and have a low education level (all *P* < 0.001) (Supplementary Table 4).

### Stressful life events

Approximately half (48.4%) of the children had experienced one or more SLEs occurring before the age of 24 months. Relocation of the family, tension from work, and physical health problems of someone close to the family were the three most frequent SLEs. Divorce, problems within the relationship of the parents, and financial problems were the three events that caused the most tension (Supplementary Table 1).

In this study, about 20% of all children (16.7%, 16.8%, and 23.9% at 24, 36, and 45 months, respectively) were at risk of psychosocial problems; less than 10% had poor general health (7.8%, 6.9%, and 7.2%, respectively). Table 2 shows that psychosocial health at each time point was different among the three subgroups by the frequency of SLEs (*P* < 0.05), whereas general health was different only at 45 months (*P* < 0.001). In comparison between children with high-tension SLEs experience and those with low-tension SLEs experience, the former children were more likely to have psychosocial problems at 36 and 45 months (*P* < 0.05) and poor general health at 24, 36, and 45 months (*P* < 0.05).

**Table 2:** Stressful life events and child health at the age of 24, 36, and 45 months (*n* = 1431)

Variables	No life events (*n* = 739)	Frequency of SLEs		*P* value	Overall tension of SLEs			*P* value
		1–2 SLEs (*n* = 546)	> 2 SLEs (*n* = 146)		No life events	Low (*n* = 353)	High (*n* = 339)	
Psychosocial problems								
24 mon, at risk	110 (14.9)	93 (17.0)	36 (25.0)	0.029	–	65 (18.4)	64 (19.0)	0.846
36 mon, at risk	85 (12.3)	94 (18.6)	44 (32.1)	< 0.001	–	58 (17.7)	80 (25.4)	0.016
45 mon, at risk	100 (18.3)	116 (26.9)	43 (40.6)	0.006	–	64 (23.4)	95 (36.0)	0.001
General health								
24 mon, poor	56 (7.6)	39 (7.2)	16 (11.0)	0.140	–	14 (4.0)	41 (12.1)	< 0.001
36 mon, poor	46 (6.7)	32 (6.3)	14 (10.1)	0.113	–	17 (5.1)	29 (9.1)	0.045
45 mon, poor	37 (6.7)	22 (5.0)	20 (18.7)	< 0.001	–	13 (4.7)	29 (10.8)	0.007

Data presented as *n* (%)

Risk of psychosocial problems was measured by the Brief Infant–Toddler Social and Emotional Assessment at 24 months of age and by the Strengths and Difficulties Questionnaire at 36 and 45 months

Poor general health was measured by General Health subscale of Child Health Questionnaire at the age of 24 months, 36 months and 45 months

*SLE* Stressful life event

*P* values are based on Chi-square test

### Stressful life events and health

Table 3 presents the results of GEE-based regressions regarding the association between SLEs occurring before the age of 24 months and health outcomes across 24–45 months. Data in the left two columns show associations between SLEs and psychosocial health by gender. Compared to boys without SLEs experience, boys who experienced > 2 SLEs (OR = 2.10, 95% CI 1.36–3.24) or high-tension SLEs (OR = 1.47, 95% CI 1.06–2.03) had higher odds of being at risk of psychosocial problems. Compared to girls without SLEs experience, the risk of psychosocial problems increased to almost three times among girls who experienced > 2 SLEs (OR = 3.31, 95% CI 2.05–5.35) or a high level of tension (OR = 3.01, 95% CI 2.07–4.39). Moreover, girls who experienced 1–2 events (OR = 2.15, 95% CI 1.54–3.00) and low-tension SLEs (OR = 1.90, 95% CI 1.31–2.74) also had higher odds of being at risk of psychosocial problems than girls who experienced no SLEs.

**Table 3:** Associations between experiencing life events before age 24 months and the risk of psychosocial problems at the age of 24–45 months (*n* = 1431)

Variables	Risk of psychosocial problems		Poor general health
	Boys OR (95% CI)	Girls OR (95% CI)	Total OR (95% CI)
Number of SLEs			
No life event	Ref	Ref	Ref
1–2 SLEs	1.13 (0.84–1.51)	2.15 (1.54–3.00)^†^	0.89 (0.63–1.25)
> 2 SLEs	2.10 (1.36–3.24)^†^	3.31 (2.05–5.35)^†^	1.96 (1.21–3.18)*
Overall experienced tension from SLEs			
No events	Ref	Ref	Ref
Low	1.13 (0.81–1.58)	1.90 (1.31–2.74)^†^	0.63 (0.40–0.99)*
High	1.47 (1.06–2.03)*	3.01 (2.07–4.39)^†^	1.60 (1.12–2.28)*

Risk of psychosocial problems was measured by the Brief Infant–Toddler Social and Emotional Assessment at 24 months and by the Strengths and Difficulties Questionnaire at 36 and 45 months

Poor general health was measured by General Health subscale of Child Health Questionnaire at child aged 24 months, 36 months and 45 months

Results presented by child gender due to the interaction effect of SLEs and gender in association of being at risk of psychosocial problems

*OR* odds ratio, *CI* confidence Interval, *SLE* stressful life event, *Ref* reference

*P < 0.05, †*P* < 0.001

Experiencing > 2 SLEs (OR = 1.96, 95% CI 1.21–3.18) or high-tension SLEs (OR = 1.60, 95% CI 1.12–2.28) was associated with higher odds of being in poor general health. However, experiencing low-tension SLEs was associated with lower odds of being in poor general health (OR = 0.63, 95% CI 0.40–0.99) (Table 3).

Supplementary Table 3 shows that the highest risk for psychosocial problems presented at 45 months after high-tension SLEs occurred before 24 months (OR = 2.52, 95% CI 1.80–3.55). However, both low- and high-tension SLEs were associated with a higher risk of psychosocial problems at 36 months (low: OR = 1.55, 95% CI 1.07–2.24, high: OR = 2.44, 95% CI 1.73–3.45).

## Discussion

This study aimed to examine associations between SLEs occurring before 24 months of age and the psychosocial and general health of children across 24, 36, and 45 months. The results indicated that experiencing > 2 SLEs or high-tension SLEs was associated with higher odds of lower psychosocial health and general health from 24 to 45 months. Moreover, only girls showed increased odds of lower psychosocial health and general health after experiencing 1–2 SLEs or low-tension SLEs.

### Stressful life events and psychosocial health

Our findings that the experience of SLEs was associated with psychosocial problems were in line with previous studies conducted in children and adolescents [6, 7]. Moreover, we observed that girls who experienced > 2 SLEs had an increased risk of psychosocial problems compared to girls who experienced 1–2 SLEs. In both boys and girls, the risk of psychosocial problems was different between experiencing 1–2 and experiencing > 2 SLEs. Children who experience one life event are more likely to experience other adversities [7]. These findings are valuable in stressing the accumulative effect of SLEs on children’s psychosocial health.

In addition to the frequency of SLEs, the impact of low-tension SLEs and that of high-tension SLEs were different for girls and boys. High-tension SLEs showed a larger impact on the psychosocial health of girls than low-tension SLEs. In boys, only high-tension SLEs increased the risk of psychosocial problems significantly. Children under the age of three years are dependent upon the care of adults, and tension experienced or presented by their parents could impact children’s overall development [9]. The presented findings support the use of a more comprehensive SLE evaluation, including an assessment of experienced tension in addition to the mere frequency of SLEs.

### Stressful life events and general health

To our knowledge, this study is the first to examine the association between SLEs and general health in preschool children. Findings regarding SLEs (> 2 SLEs and high-tension SLEs) and general health were in line with previous studies conducted in adolescents [16, 27, 28]. The findings of a 12-year follow-up study revealed that stressful life events experienced by mothers in the year after childbirth could increase odds of poor general health during a child’s adolescence [28]. However, we observed that children with low-tension SLEs had a lower risk of poor general health than children without such experience. Certain SLEs measured in our study might partly explain this finding. For example, relocation of the family hypothetically may lead to a better residence, which could benefit children’s general health and well-being. However, this is a hypothesis, and the possibility of chance finding could not be ruled out. This was especially true since the differences were insignificant in the multiple comparison test (*P* > 0.05). Moreover, it needs to be mentioned that there was a low percentage of children with poor general health in this relatively large sample, which could have impacted the findings. Future research is recommended to examine the associations in a larger population.

### Gender differences

Gender differences in SLEs and psychosocial health have been widely discussed but with contrary findings. In the review by Grant, 39 of 68 studies reported the moderation effect of gender in the association between stress and child psychosocial health. In adults, women were more strongly associated with depression onset after exposure to SLEs than men [2, 29]. However, the higher prevalence of depression was not related to the different frequencies of SLEs reported or different tensions caused by SLEs [29]. There have been very few studies addressing gender differences in SLEs and general health. Our findings were in line with the longitudinal study that reported insignificant gender differences in the association between SLEs and overall well-being measured by the KIDSCREEN-27 in children and adolescents aged 8–18 years [30].

Moreover, a higher risk of psychosocial problems increased for girls after experiencing > 2 SLEs or high-tension SLEs compared to boys with the same experience. The stronger association between SLEs and psychosocial health in girls was consistent with studies that observed gender differences [16, 30]. Mechanisms underlying the child gender difference and potential intervention to reduce gender inequality in young children need to be explored in future research.

### Stressful life events

This study assessed the overall tension of SLEs in addition to the frequency of SLEs, considering that individuals might react differently to the same SLEs [2, 31]. In addition, multiple categorical settings (i.e., 0/1–2/ > 2 events and no event/low/high tension) enabled us to explore the accumulation effect of SLEs and whether the perceived tension level impacts [2, 5]. The SLEs assessed in this study might be considered low risk compared to adverse childhood experiences (ACEs) like physical, emotional, and sexual abuse but normative across early childhood [2]. Nevertheless, both the frequency and overall tension of SLEs before the age of 24 months were associated with lower psychosocial health and general health. Therefore, youth health care professionals may pay attention to the impact and tension caused by these SLEs.

### Strengths and limitations

There are strengths of this study. First, this longitudinal study with repeated measurements of outcomes contributes to the evidence base, also using a comprehensive measurement of SLEs [4, 13]. Moreover, age-specific tools have been used to assess psychosocial problems, including the BITSEA at 24 months and the SDQ at 36 and 45 months. However, there are some limitations. First, 12 SLEs occurring before children aged 24 months were parent-reported and might be prone to recall bias. In addition, future studies are recommended to include multiple types of SLEs. Second, although age-specific tools improved accuracy in measuring psychosocial health in preschool children (2–4 years), comparability between tools must be considered when their outcomes are treated as repeated measurements. The total SDQ score was positively correlated with the BITSEA Problem score and negatively correlated with the Competence score in a Turkish version used in previous research [32]. More information regarding comparability is needed to avoid misclassification of psychosocial problems. Third, potential confounders reported in previous studies cannot be ruled out as data unavailability, for example, parental mental health [33].

In conclusion, this study investigated the impact of SLEs in early childhood. Experiencing SLEs occurring before 24 months was associated with lower psychosocial health and general health in preschool children. More pronounced associations were observed in girls. The findings emphasized that SLEs at a young age impacted the psychosocial and general health of children. Attention and support for families is needed to minimize the impact in families experiencing these SLEs, particularly in girls.

## Supplementary Information

Below is the link to the electronic supplementary material.

Supplementary file1 (DOCX 52 KB)

## Data Availability

Data are available upon request by contacting the corresponding author.
