# Supplementary material for: Stressful life events, psychosocial health and general health in preschool children before age 4
Source: World J Pediatr. 2022 Nov 17;19(3):243–50. doi: 10.1007/s12519-022-00639-w (PMC9974714; doi:10.1007/s12519-022-00639-w)
Supplement: Supplementary file 1 — Supplementary file1 (DOCX 52 KB) [file 12519_2022_639_MOESM1_ESM.docx]

## *Supplementary material*

Missing data on stressful life events (n = 86)

Parents with available data of stressful life event before child age 24 months

(n = 2219)

Final sample for analyses

(n = 1431)

Final sample for analyses

(n=1470)

Parents who completed only one measurement of any outcome

(n = 639)

### Supplementary Fig 1. Population for analyses

Excluded for analyses:

Missing covariates data (n = 149)

The second child of a twin (n = 18)

Parents who completed at least two measurements of each outcome at child age 24, 36, and 45 months

(n = 1580)

Parents who complete the baseline questionnaire at child age 24 months

(n = 2305)

Supplementary Table 1. Tension score for individual stressful life event (n = 692)

|  |  |  | Tension score | |  |
| --- | --- | --- | --- | --- | --- |
|  | N^1^ | A little (n) | Somewhat  (n) | A lot  (n) | Mean ± SD |
| Relocation of the family | 247 | 148 | 70 | 29 | 1.3 ± 0.6 |
| Relocation of someone close to the child | 11 | 7 | 3 | 1 | 1.2 ± 0.4 |
| Tensions at work of one of the parents, felt at home | 229 | 55 | 130 | 44 | 1.7 ± 0.6 |
| Financial problems | 54 | 10 | 24 | 20 | 1.9 ± 0.6 |
| Quarrels with neighbours/friends/ acquaintances/family | 42 | 7 | 23 | 12 | 1.8 ± 0.6 |
| Victim of fire or burglary | 21 | 11 | 6 | 4 | 1.4 ± 0.5 |
| Physical health problems of someone close to the family | 201 | 82 | 87 | 32 | 1.6 ± 0.7 |
| Mental health problems of someone close to the family | 106 | 32 | 45 | 29 | 1.7 ± 0.7 |
| Death of someone close to the family | 138 | 73 | 53 | 12 | 1.3 ± 0.6 |
| Problems within relationship of the parents | 69 | 13 | 31 | 25 | 2.0 ± 0.7 |
| Divorce | 26 | 8 | 9 | 9 | 2.1 ± 0.8 |
| Unemployment of one of the parents | 140 | 69 | 46 | 25 | 1.4 ± 0.6 |

Note: SD = Standard Deviation.
^1^ Frequency of life event reported among the total participants (n = 692).

Supplementary Table 2 Total frequency of SLEs and tension score frequencies (n = 1431)

|  |  |  | Tension score |  |
| --- | --- | --- | --- | --- |
| Frequency of SLEs | Total  N (%) | A little  n (%) | Somewhat  n (%) | A lot  n (%) |
| No event | 739 (51.6) | - | - |  |
| 1-2 SLEs | 546 (38.2) | 319 (90.1) | 196 (75.7) | 31 (39.2) |
| >2 SLEs | 146 (10.2) | 35 (9.9) | 63 (24.3) | 48 (60.8) |
| Total SLEs | 1431(100.0) | 354 (24.7)^1^ | 259 (18.1)^2^ | 79 (5.5)^2^ |

SLE = Stressful Life Event

1 Comprises the group of ‘low tension’ on the ‘Overall tension’ variable.
2 Comprises the group of ‘high tension’ on the ‘Overall tension’ variable.

Supplementary Table 3. Associations between experiencing a life event before age 24 months and being at risk of psychosocial problems at age 24, 36, and 45 months (n=1431).

|  | Risk of psychosocial problems^1^ | | |
| --- | --- | --- | --- |
|  | 24 months  (N = 1428) | 36 months  (N = 1331) | 45 months  (N = 1085) |
| Overall tension of SLEs^2^ |  |  |  |
| No events | Ref | Ref | Ref |
| Low | 1.34 (0.94-1.90) | 1.55 (1.07-2.24) * | 1.38 (0.96-1.97) |
| High | 1.32 (0.93-1.87) | 2.44 (1.73-3.45) ^†^ | 2.52 (1.80-3.55) ^†^ |

^1^ Risk of psychosocial problems was measured by the Brief Infant–Toddler Social and Emotional Assessment at 24 months and by the Strengths and Difficulties Questionnaire at 36 and 45 months.

^2^ Generalized Estimating Equations model: overall tension of SLEs as independent variable and adjusted for child gender, child ethnic background, maternal education levels and single-parent family.

* *P* < 0.05; † *P* < 0.001.

Supplementary Table 4. sensitivity analysis (N = 2305).

|  |  | Included in analyses | | *P*-value |
| --- | --- | --- | --- | --- |
|  |  | No  (n=874) | Yes  (n=1431) |  |
| Child gender, boy [n (%)] |  | 447 (52.0) | 712 (49.8) | 0.294 |
| Child ethnic background, non-Dutch [n (%)] |  | 310 (42.7) | 271 (18.9) | **<0.001** |
| Maternal ethnic background, non-Dutch [n (%)] |  | 366 (48.3) | 337 (24.1) | **<0.001** |
| Paternal ethnic background, non-Dutch [n (%)] |  | 323 (44.0) | 307 (21.9) | **<0.001** |
| Maternal educational level, low [n (%)] |  | 112 (14.9) | 80 (5.6) | **<0.001** |
| Paternal educational level, low [n (%)] |  | 140 (19.5) | 151 (10.8) | **<0.001** |
| Single-parent family, yes [n (%)] |  | 132 (16.4) | 71 (5.0) | **<0.001** |

Note. Number of missing: child gender=14, child ethnic background=148, maternal ethnic background=152, paternal ethnic background=168, maternal educational level=121, paternal educational level=196, family structure=69.

Significant p-values (p <0.05) were in bold. *P* values are based on Chi-square test.
